# AKT-induced PKM2 phosphorylation signals for IGF-1-stimulated cancer cell growth

**DOI:** 10.18632/oncotarget.10179

**Published:** 2016-06-20

**Authors:** Young Soo Park, Dong Joon Kim, Han Koo, Se Hwan Jang, Yeon-Mi You, Jung Hee Cho, Suk-Jin Yang, Eun Sil Yu, Yuri Jung, Dong Chul Lee, Jung-Ae Kim, Zee-Yong Park, Kyung Chan Park, Young Il Yeom

**Affiliations:** ^1^ Biotherapeutics Translational Research Center, Korea Research Institute of Bioscience & Biotechnology (KRIBB), Daejeon 34141, Korea; ^2^ Personalized Genomic Medicine Research Center, Korea Research Institute of Bioscience & Biotechnology (KRIBB), Daejeon 34141, Korea; ^3^ School of Life Sciences, Gwangju Institute of Science and Technology, Gwangju 61005, Korea; ^4^ Department of Pathology, Asan Medical Center, University of Ulsan College of Medicine, Seoul 05505, Korea; ^5^ Department of Functional Genomics, University of Science and Technology, Daejeon 34113, Korea

**Keywords:** PKM2, AKT, IGF-1, phosphorylation of PKM2, STAT5

## Abstract

Pyruvate kinase muscle type 2 (PKM2) exhibits post-translational modifications in response to various signals from the tumor microenvironment. Insulin-like growth factor 1 (IGF-1) is a crucial signal in the tumor microenvironment that promotes cell growth and survival in many human cancers. Herein, we report that AKT directly interacts with PKM2 and phosphorylates it at Ser-202, which is essential for the nuclear translocation of PKM2 protein under stimulation of IGF-1. In the nucleus, PKM2 binds to STAT5A and induces IGF-1-stimulated cyclin D1 expression, suggesting that PKM2 acts as an important factor inducing STAT5A activation under IGF-1 signaling. Concordantly, overexpression of STAT5A in cells deficient in PKM2 expression failed to restore IGF-induced growth, whereas reconstitution of PKM2 in PKM2 knockdown cells restored the IGF-induced growth capacity. Our findings suggest a novel role of PKM2 in promoting the growth of cancers with dysregulated IGF/phosphoinositide 3-kinase/AKT signaling.

## INTRODUCTION

Insulin-like growth factors (IGFs) promote cell proliferation and inhibit apoptosis [1–3]. IGFs are also associated with the pathological development of many types of tumors, including breast cancer [4–6]. Indeed, a high serum level of IGFs has been reported as a risk factor for pancreatic, lung, colorectal, and breast cancers [7–9]. Low levels of IGFBP-3, a negative regulator of IGF signals, have been associated with the development of malignancy and the malignant phenotype [7, 10]. In addition, increased expression of IGF-1 receptor (IGF-1R) has been detected in a subset of patients with lung cancer and other cancers [11, 12]. IGF-1R contains a tyrosine kinase domain that is responsible for activating the phosphoinositide 3-kinase (PI3K)/AKT/mTOR and Ras/Raf/mitogen-activated protein kinase pathways that promote cell growth, transformation, migration, and survival [13–15]. Based on the reported associations between IGF signaling and the risk of cancer development, the IGF pathway has been suggested as a promising therapeutic target for many types of cancers. However, the regulatory mechanisms underlying the influence of IGF signaling on cancer development and progression remain to be fully elucidated.

During tumorigenesis, cellular metabolism is altered to prime cells for transformation in a manner that facilitates their anabolic growth and survival [16, 17]. Recently, increased research interest has been directed to studying the mechanism of the metabolic alterations observed during malignant transformation [18]. Cancer cells typically exhibit a high rate of glycolysis and mainly produce lactic acid from pyruvate oxidation, even under aerobic conditions (the so-called “Warburg effect”) [18, 19]. Pyruvate kinases are glycolytic enzymes that convert phosphoenolpyruvate to pyruvate in the final step of glycolysis [20]. Pyruvate kinase muscle type 2 (PKM2) is one of four pyruvate kinase isozymes and has been shown to promote cancer cell metabolism and growth, especially in highly glycolytic cancer cells [21, 22]. Concordantly, PKM2 is highly expressed in many cancers, including lung cancer, and is associated with poor prognosis in cancer patients [23, 24].

Recently, there have been many reports on the modifications of PKM2 induced by tumor microenvironmental factors. Fibroblast growth factor receptor 1 (FGFR1) induces the phosphorylation of PKM2 at tyrosine (Tyr) residue 105, resulting in a decrease in lactate production while promoting the induction of tumor cell growth [25]. Stimulation of human glioblastoma multiforme (GBM) cells with epidermal growth factor (EGF) causes phosphorylation of PKM2 at serine (Ser) residue 37 and translocation of the PKM2 protein to the nucleus. This in turn results in an increase of cyclin D1 (CCND1) and c-Myc expression, thereby promoting tumor cell growth and brain tumorigenesis [25–27]. In addition, under hypoxic conditions, hydroxylated PKM2 binds with hypoxia inducible factor 1 (HIF1)-α to increase its activity [28]. In the present study, we evaluated the role of PKM2 in promoting the growth of cancers associated with aberrant IGF-1 signaling.

## RESULTS

### IGF-1-induced cell growth is associated with the expression of PKM2

**Figure 1:**
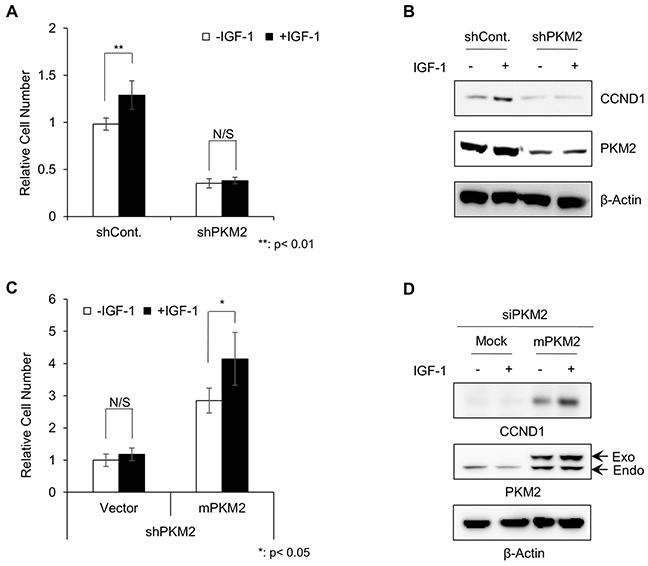
H1299 lung cancer cells grew in a PKM2-dependent manner under IGF-1 stimulation **A.** Results of cell proliferation assay with treatment of control shRNA or PKM2 shRNA under IGF-1 stimulation for 5 days. The cell numbers are presented relative to the number of IGF-untreated control shRNA cells. *p-value < 0.05, SD (n=3). **B.** Western blot analysis of CCND1 expression in control shRNA and PKM2 shRNA stable cells. After being starved with 0.5% FBS medium for 24 h, the cells were treated with or without IGF-1 (200 ng/ml) for 24 h in serum-free media. **C.** Cell proliferation assay of PKM2-reconstituted PKM2 shRNA stable cells under IGF-1 stimulation for 5 days. The cell numbers are presented relative to the number of IGF-untreated control shRNA cells. *p-value < 0.05, SD (n=3). **D.** Western blot analysis of CCND1 induction by PKM2 reconstitution in PKM2 knockdown cells.

We examined the involvement of PKM2 on the proliferation of NCI-H1299 lung cancer cells in response to IGF-1, given that high IGF-1 serum levels have been reported as a risk factor for lung cancer [7]. IGF-1 significantly induced the growth of NCI-H1299 cells compared to non-treated control cells. However, stable knockdown of PKM2 using a PKM2-specific small hairpin RNA (shRNA) vector (Supplementary Figure S1) abolished the IGF-1-induced growth in these cells (Figure 1A). In parallel, the IGF-1-induced expression of CCND1, a marker of IGF-1 signaling activation [29], was also disrupted in the PKM2 knockdown cells (Figure 1B).

To verify that the loss of IGF-1-induced proliferation in PKM2-deficient cells was due to the depletion of PKM2 function, we reconstituted the PKM2 function in the PKM2 knockdown cells by transduction of an expression vector carrying a gene encoding a mouse PKM2 protein (mPKM2), and investigated the responses to IGF-1 stimulation. Indeed, exogenous PKM2 significantly restored IGF-1-induced cell proliferation (Figure 1C, Supplementary Figure S2), which was in parallel with the recovery of CCND1 induction (Figure 1D). These results indicate that PKM2 may have important roles in cancer progression by promoting IGF-1-induced cell proliferation.

### PKM2 physically binds with AKT1

**Figure 2:**
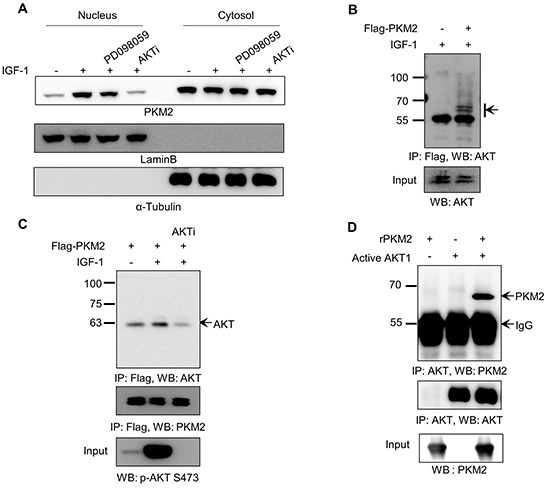
AKT1 directly binds to PKM2 **A.** AKT signal is required for the nuclear translocation of PKM2 under IGF-1 stimulation. H1299 cells were treated with or without IGF-1 (200 ng/ml) for 12 h, and were then treated with PD098059 (10 μM) or AKT inhibitor (5 μM) 30 min before IGF-1 treatment. Lamin B was used as a nuclear marker and α-tubulin was used as a cytosolic marker. **B.** Identification of endogenous AKT as a binding partner of PKM2. H1299 cells transfected with mock or Flag-PKM2 vector were treated with IGF-1 as described in the legend to Figure 1. Cell lysates were subjected to an immunoprecipitation assay using anti-Flag antibody beads. PKM2 and its binding partners were eluted with Flag-peptide (0.1 mg/ml). The eluted fraction was applied to western blot analysis with anti-AKT antibody. The arrow indicates immuno-reactive bands in the AKT size range. **C.** Binding of Flag-PKM2 with activated AKT in H1299 lung cancer cells. The levels of phospho-AKT(S473) in each sample are shown in the bottom panel. **D.** PKM2 directly binds with AKT1. Recombinant PKM2 was incubated with or without recombinant active AKT1, and then the samples were subjected to an immunoprecipitation assay with anti-AKT antibody. The immunoprecipitated samples were then applied to western blot analysis.

Previous reports demonstrated that PKM2 usually localizes to the nucleus after its modification in the cytosol in response to specific extracellular signals [30]. Thus, we next investigated the potential mechanism of IGF-1-induced cell proliferation, by verifying whether IGF-1 signaling can modify and subsequently induce the translocation of PKM2 protein into the nucleus. As shown in Figure 2A, IGF-1 treatment increased the nuclear level of PKM2. To identify the IGF-1 pathway signal(s) responsible for this translocation, we treated cells with IGF-1 along with pharmacological inhibitors of MEK and AKT (PD098059; MEK inhibitor, AKTi; AKT inhibitor), which are the two major downstream kinases of the IGF-1 pathway. AKT inhibition specifically suppressed the IGF-1-induced nuclear localization of PKM2, whereas MEK inhibition had no effect (Figure 2A). This result suggests that AKT, or its downstream signals, may modulate the intracellular behavior of PKM2 protein under IGF-1 signaling. To verify this, we examined the possible binding between PKM2 and AKT by immunoprecipitating Flag-tagged PKM2 from the lysates of transfected NCI-H1299 cells after treatment with IGF-1. Western blot analysis using anti-AKT antibody clearly indicated that endogenous AKT protein co-immunoprecipitated with PKM2 protein (Figure 2B). Furthermore, although IGF-1 treatment significantly enhanced the binding between the two proteins, AKTi treatment effectively disrupted the co-immunoprecipitation reaction in conjunction with inhibition of AKT activation (Figure 2C). These results indicate that PKM2 and AKT can physically bind in an IGF-1-dependent manner, and that their binding requires AKT activation. The binding between PKM2 and AKT was further validated by an *in vitro* binding assay using recombinant His-tagged PKM2 and active AKT1 proteins (Figure 2D).

### AKT1 phosphorylates PKM2 at serine residue(s)

**Figure 3:**
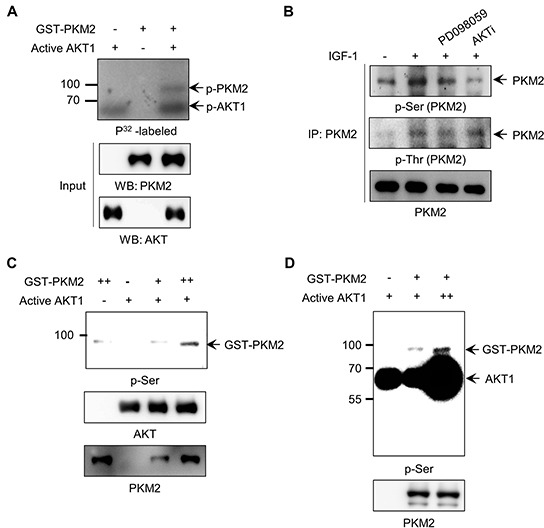
AKT1 phosphorylates PKM2 at serine residues **A.** *In vitro* kinase assay for PKM2 phosphorylation by AKT using [γ-^32^P]ATP. **B.** AKT signal-induced PKM2 phosphorylation at serine residue(s) in H1299 cells stimulated with IGF-1. **C-D.** Verification of PKM2 phosphorylation at serine residue(s) by AKT via an *in vitro* kinase assay with anti-Ser antibody using different amounts of PKM2 (C) or active AKT (D) proteins.

PKM2 proteins are typically phosphorylated at specific Ser or Tyr residues under growth-promoting conditions induced by EGFR or FGFR activation [25, 27]. Based on the results described above, we hypothesized that IGF-1-activated AKT might directly phosphorylate PKM2. To verify this possibility, we performed an *in vitro* kinase assay for glutathione S-transferase (GST)-tagged PKM2 protein using active recombinant AKT and [γ-P^32^]ATP. We observed a prominent phosphor-protein band corresponding to the estimated molecular mass of GST-PKM2 (95 kDa) only in the reaction containing both GST-PKM2 and active AKT proteins (Figure 3A). Since AKT is a Ser/Thr kinase, we next determined which residue(s) of PKM2 are specifically phosphorylated by AKT1, using antibodies for phospho-Ser or phospho-Thr. We also used the AKT and MEK inhibitors to discriminate between the AKT- or ERK-induced phosphorylation of PKM2, as previously reported [27]. The results showed that PKM2 was phosphorylated at both Ser and Thr residues in response to IGF-1, and that AKT is responsible for the majority of the Ser phosphorylation, whereas MEK is responsible for a larger part of the Thr phosphorylation (Figure 3B). We also confirmed the Ser phosphorylation of PKM2 by AKT1 using exogenously expressed Myc-PKM2 protein (Supplementary Figure S3).

We further verified these results with an *in vitro* kinase assay using recombinant proteins. A phospho-protein band was identified with anti-p-Ser antibody at the molecular mass estimated for GST-PKM2 in the AKT1-containing reaction (Figure 3C, lane 4), in contrast to a faint non-specific band observed in the control lane lacking active AKT1 (Figure 3C, lane 1). In addition, after incubation of the recombinant GST-PKM2 protein with different amounts of active AKT1 in the presence of ATP, the intensity of the phosphor-Ser band of PKM2 protein correlated with the amount of AKT1 protein added to the reactions (Figure 3D). These results suggest that AKT1 is a *bona fide* protein kinase that phosphorylates PKM2 at Ser residues under IGF-1 signaling.

### AKT1 phosphorylation of the PKM2 Ser-202 residue is required for its nuclear localization under IGF-1 signaling

**Figure 4:**
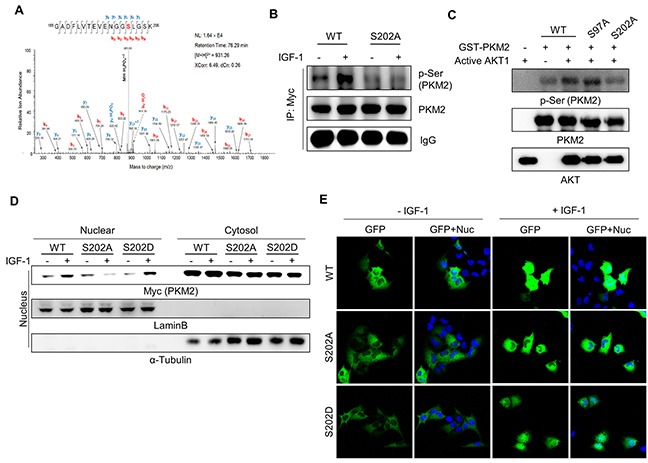
AKT phosphorylates PKM2 at Ser-202, which is required for the nuclear localization of PKM2 **A.** Mass spectrometry analysis revealed AKT phosphorylation of PKM2 at the Ser-202 residue. An *in vitro* kinase assay was performed with GST-PKM2 (5 μg) and active AKT1, and then the phosphorylated sites of PKM2 were analyzed by LC-MS/MS. **B.** Validation of phosphorylation of PKM2 at Ser-202 using the S202A mutant PKM2. H1299 cells were transfected with the MYC-tagged wild-type (WT) or MYC-tagged S202A mutant PKM2 vector and then treated with IGF-1 (200 ng/ml) for 30 min. The cell lysates were subjected to an immunoprecipitation assay using anti-MYC antibody. **C.** Validation of the phosphorylation of PKM2 at Ser-202 via an *in vitro* kinase assay using WT or mutant PKM2 proteins. **D-E.** Ser-202 phosphorylation of PKM2 is required for the nuclear localization of PKM2. The nuclear localization of PKM2 was determined by western blot analysis following nuclear fractionation assay (D) and by imaging the fluorescence of GFP-fused wild-type or S202 mutant PKM2 proteins. Lamin B was used as a nuclear marker and α-tubulin was used as a cytosolic marker (D). Green (for GFP) and blue (for DAPI staining) colors represent GFP-fused PKM2 proteins or the nucleus, respectively (E).

To identify the specific Ser residue(s) of PKM2 phosphorylated by AKT, we performed liquid chromatography-tandem mass spectrometry (LC-MS/MS) analysis for recombinant GST-PKM2 subjected to an *in vitro* kinase reaction in the presence of recombinant active AKT1 protein. This analysis revealed that PKM2 is phosphorylated at Ser-37, Ser-97, and Ser-202. Among these sites, phosphorylation at Ser-202 was detected with the highest number of MS/MS spectrum (Figure 4A), suggesting Ser-202 as the primary site for the AKT1-induced phosphorylation of PKM2. To verify the biological relevance of the Ser-202 phosphorylation *in vivo*, we performed a cell-based assay using pcDNA-Myc/His-PKM2 vectors encoding wild-type *PKM2* or a mutant in which the Ser-37, -97, or -202 residue was converted to alanine (PKM2(S37A), PKM2(S97A), or PKM2(S202A), respectively). Myc-tagged PKM2 proteins were immunoprecipitated from the lysates of expressing the recombinant PKM2 proteins, and analyzed by western blotting using anti-p-Ser antibody to assess the AKT1-induced Ser phosphorylation of recombinant PKM2 proteins under IGF-1 stimulation. The level of Ser phosphorylation of wild-type PKM2 increased in the presence of IGF-1, while that of PKM2(S202A) seemed to have lost the IGF-1 dependency (Figure 4B). However, the S37A mutant of PKM2 still exhibited the wild-type pattern of IGF-1-induced Ser phosphorylation (Supplementary Figure S4). These results indicate that the Ser-202 residue is the major site phosphorylated by AKT under IGF-1 stimulation.

To further confirm the AKT phosphorylation of PKM2 at Ser-202, we performed an *in vitro* kinase assay using recombinant GST-PKM2 proteins, followed by western blot analysis using anti-p-Ser antibody. The phospho-Ser signal intensity for GST-PKM2(S202A) was much weaker than that of the wild-type (GST-PKM2(WT)) or the Ser-97 mutant (GST-PKM2(S97A)) proteins (Figure 4C). These results collectively confirmed that Ser-202 is the genuine phosphorylation site of PKM2 by AKT.

Post-translational modification-induced transloca-tion of PKM2 to the nucleus is often associated with enhanced cell proliferation and cancer malignancy [26, 28, 31]. Thus, we investigated whether Ser-202 phosphorylation is essential for the nuclear localization of PKM2. In contrast to the wild-type PKM2 protein, which exhibited an IGF-1-induced nuclear translocation pattern, the nuclear level of mutant PKM2(S202A) was not increased by IGF-1 treatment (Figure 4D). This was more clearly confirmed by using GFP-fused PKM2 constructs (Figure 4E, Supplementary Figure S6), suggesting that Ser-202 phosphorylation is required for the IGF-1-stimulated nuclear localization of PKM2. However, the phosphorylation mimetic mutant of PKM2 Ser-202, PKM2(S202D), was translocated to the nucleus only in the presence of IGF-1 stimulation but not on its own (i.e., it was not translocated in the absence of IGF-1 stimulation).

### PKM2 physical binds with STAT5A via S202 phosphorylation

**Figure 5:**
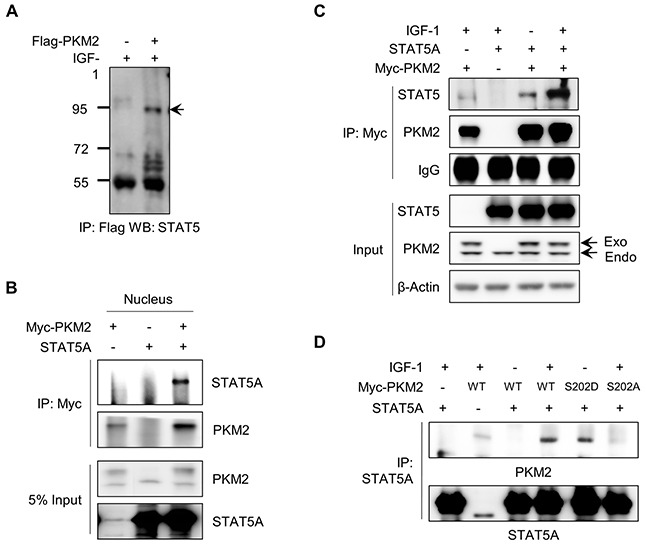
IGF-1-mediated PKM2 binding with STAT5A **A.** PKM2 binds to STAT5A. H1299 cells were transfected with vector or Flag-PKM2 and then treated with IGF-1 for 30 min. The cell lysates were subjected to an immunoprecipitation assay using anti-Flag antibody and then western blot analysis was carried out using STAT5A antibody. **B.** The binding between PKM2 and STAT5A was identified in the nuclear fraction of the cells treated with IGF-1. **C.** PKM2 binds with STAT5A in an IGF-1 stimulation-dependent manner. The cells transfected with STAT5A and/or PKM2 vectors are indicated. **D.** Phosphorylation of PKM2 at Ser-202 is required for the binding of PKM2 with STAT5A. Cells transfected with the indicated PKM2 vectors were treated with IGF-1 for 30 min. Cells were harvested and an immunoprecipitation assay was performed using STAT5A-specific antibody. Western blotting was performed with anti-PKM2 antibody.

The STAT5-induced increase of CCND1 expression is one of the crucial effector responses in IGF-1-induced cell proliferation [29]. We investigated whether PKM2 can increase CCND1 expression under IGF stimulation and subsequent cell growth via tethering the STAT5 activity. We first conducted an immunoprecipitation assay to examine whether PKM2 physically binds with STAT5 in the presence of IGF-1 using NCI-H1299 cells expressing Flag-PKM2. Endogenous STAT5 proteins specifically immunoprecipitated from the Flag-PKM2-expressing cell lysate (Figure 5A). A co-immunoprecipitation assay in nuclear extracts further confirmed the intracellular location of the binding between PKM2 and STAT5A in the nucleus (Figure 5B).

Next, we investigated whether the PKM2–STAT5A binding requires IGF-1 signaling. We performed a coimmunoprecipitation assay using anti-Myc antibody with the lysates of the cells expressing Myc/His-PKM2 and/or STAT5A with or without IGF-1 treatment. As a result, STAT5A protein was co-immunoprecipitated with Myc-PKM2 in the IGF-1-treated cells (Figure 5C), suggesting that the binding between PKM2 and STAT5 may require the IGF-1-induced Ser-202 phosphorylation of PKM2. We then examined the relevance of the Ser-202 phosphorylation of PKM2 for its binding with STAT5 using wild-type and mutant PKM2 (S202D or S202A). As shown in Figure 5D, wild-type PKM2 was found to bind with STAT5A when the cells were treated with IGF-1, but PKM2(S202A) failed to bind with STAT5A. In addition, the phosphorylation mimetic mutant of PKM2 (S202D) bound with STAT5A even without IGF-1 stimulation.

### PKM2 promotes cancer cell growth via STAT5 activation under IGF-1 stimulation

**Figure 6:**
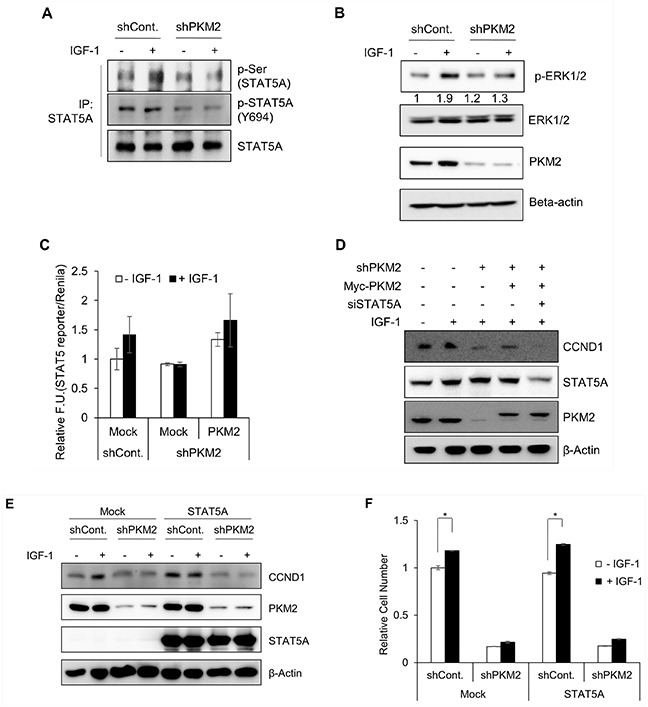
PKM2-dependent STAT5A transcriptional activity **A–B.** PKM2 influences STAT5A phosphorylation in H1299 cells. Control shRNA and PKM2 shRNA stable cells were treated with or without IGF-1 (200 ng/ml) for 30 min. (A) An immunoprecipitation assay was performed using STAT5A-specific antibody, and the levels of phospho-STAT5A were determined with the indicated antibodies. (B) The levels of phospho-ERK1/2 were determined in the cell lysates. Quantitative results of the band intensity estimated using the Image J (NIH) software program are shown under western blotting data. **C.** PKM2 induces STAT5 activity in IGF-1-stimulated cells. STAT5 activity was determined using the pGL4.52 (luc2p/STAT5 RE/Hygro) reporter vector system. The data represent relative values of firefly luciferase activity normalized to *Renilla* luciferase activity. All data are shown as the mean ± SD of values obtained from 3 independent experiments with duplicate samples. **D.** PKM2-dependent CCND1 expression requires STAT5A expression. PKM2 expression was reconstituted with a PKM2-expressing vector in PKM2 shRNA stable cells with or without the STAT5A-specific siRNA (siSTAT5A-2) (Supplementary Figure S7). **E.** STAT5A cannot induce CCND1 expression without PKM2. Control shRNA and PKM2 shRNA cells were transfected with mock or STAT5A-expressing vector. After being stimulated with IGF-1, the cells were subjected to a western blot analysis using anti-CCND1 antibody. **F.** STAT5A could not rescue the IGF-induced cell growth without PKM2. Control shRNA or PKM2 shRNA stable cells were transfected with mock or STAT5A-expressing vector, and then treated with IGF-1 (200 ng/ml). After 5 days, the cell numbers were counted.

Finally, we investigated whether PKM2 indeed stimulates the transcriptional activity of STAT5 to increase IGF-1-induced cell proliferation. We first examined the effects of PKM2 depletion on the levels of STAT5 phosphorylation at Ser and Tyr residues. IGF-1 induced the phosphorylation of STAT5A at Ser residue(s) but not at Tyr-694 residue in control cells, whereas PKM2 depletion blocked the IGF-1-induced phosphorylation of STAT5, including the basal level of Tyr-694 phosphorylation in unstimulated conditions (Figure 6A). To identify the kinase that works with IGF-induced nuclear phospho-PKM2(S202) to induce STAT5 activation under IGF-1 stimulation, we focused on JAK2 and ERK1/2, as the kinases typically responsible for the phosphorylation of STAT5 at Tyr or Ser residues, respectively. ERK1/2 activation (i.e., phosphorylation at Thr-202/Tyr-204 residues) was increased (by about 1.9-fold) by IGF-1 treatment. By contrast, PKM2 depletion did not affect the level of phosphor-ERK1/2 in the absence of IGF-1 but significantly diminished the IGF-1-induced phosphorylation of ERK1/2 (Figure 6B, top panel), which matched the level of p-Ser-STAT5 (Figure 6A, top panel). Together with the results of the IGF-induced phosphorylation of STAT5 at Ser residue(s), these results suggest that ERK1/2 may be the kinase working with PKM2 to activate STAT5A under IGF-1 stimulation. By contrast, PKM2 depletion itself down-regulated the level of phosphor-Y694-STAT5 regardless of IGF-1stimulation. In addition, the level of phosphor-Y694-STAT5 in the both the control and PKM2-depleted cells was only slightly or not increased under IGF-1 stimulation (Figure 6A, middle panel), implying that JAK2, a Tyr kinase, may be not be the kinase responsible for the IGF-dependent activation of STAT5A.

We next investigated whether the PKM2 knockdown-mediated inhibition of STAT5A phosphor-ylation influences its transcriptional activity. The STAT5-specific reporter assay showed that PKM2 depletion blocked the IGF-induced increase in STAT5 activity. Reconstitution of PKM2 expression significantly restored the STAT5 activity in PKM2 knockdown cells (Figure 6C). The specificity of the reporter system for STAT5 activity was validated by showing that overexpression of STAT5, but not STAT3, increased both the basal level and IGF-1-induced activities of the reporter (Supplementary Figure S7). Furthermore, we determined whether PKM2 could control CCND1 gene expression and cell proliferation via promoting STAT5 activity under IGF-1 stimulation. The IGF-1-induced increase of CCND1 expression was severely disrupted by PKM2 knockdown but was significantly restored by its reconstitution with the PKM2 gene. However, the restored CCND1 expression was dramatically decreased following treatment with a STAT5A-specific siRNA (siSTAT5A-2) (Figure 6D; Supplementary Figure S8). This indicated that PKM2 might function as an upstream activator of STAT5 for CCND1 expression during IGF-1 signaling. To confirm this possibility, we investigated the direct effect of PKM2 depletion on CCND1 expression in the cells overexpressing STAT5, and found that STAT5 failed to restore the expression of CCND1 suppressed in PKM2-depleted cells regardless of the presence of IGF-1 (Figure 6E). Together with the data for PKM2-STAT5 binding, these results indicate that PMK2 promotes STAT5 activity under IGF-1 signaling. This was further supported by the result that overexpression of STAT5A could not rescue the reduction in cell growth caused by PKM2 deficiency (Figure 6F).

## DISCUSSION

IGFs, a growth factor family promoting cancer development and progression [4–6], are involved in the alteration of energy metabolism in cancer cells, specifically aerobic glycolysis [18, 32, 33]. PKM2 protein undergoes post-translational modifications in response to growth factor signals, whereby it acquires cancer-promoting functions [25, 26, 28, 31]]. These facts led us to examine the potential cancer-promoting functions of PKM2 in the IGF signaling pathway. We identified a novel phosphorylation of PKM2 at Ser-202, which is induced by AKT activation under IGF-1 stimulation. This phosphorylation was essential for the nuclear translocation of PKM2 protein, allowing it to promote cancer cell growth under IGF-1 stimulation.

However, the Ser-202 phosphorylation of PKM2 by itself is not sufficient, albeit necessary, for the nuclear localization of PKM2 under IGF stimulation, suggesting additional modification in PKM2. Phosphorylation of PKM2 at Ser-37, which was demonstrated by Yang *et al.* [26], is not a plausible mechanism for the IGF-1-induced nuclear localization of PKM2 (Figure 4D, Supplementary Figure S9). Phosphorylation of PKM2 at Thr residue(s) is another candidate mechanism for the nuclear translocation of PKM2, since IGF-1 stimulation also induced the Thr phosphorylation of PKM2 (Figure 3B). Given that the AKT inhibitor blocked the IGF-induced nuclear localization of PKM2 (Figure 2A), AKT-induced additional Ser or Thr phosphorylation(s) not identified in our LC-MS/MS analysis might be required for the flawless nuclear localization of PKM2 under IGF-1 stimulation.

Interestingly, we found that Ser-202 phosphorylation of PKM2 is required not only for the nuclear localization of PKM2 but also for its interaction with STAT5 under IGF-1 stimulation (Figure 4D, Figure 5D). This result might provide a new perspective on the signal-specific selection of binding partners for PKM2 as it exerts tumor-promoting functions in the nucleus. PKM2 protein is differentially modified by different signals, which all result in the same effect, i.e., the nuclear translocation of PKM2. However, for PKM2 to induce signal-specific phenotypes, it must be able to interact with distinct binding partners in the nucleus in response to different cell signals. Therefore, the finding that the phosphorylation of PKM2 at Ser-202 is essential for its specific interaction with STAT5 may provide an example as to how nuclear PKM2 can substantiate distinct functions in accordance with the nature of upstream signals.

Recently, the assumption that PKM2 works as a protein kinase in the nucleus was denied by Hosios et al. [34]. Coinciding with this report, we could not find any evidence that PKM2 functions as a protein kinase in the IGF-induced phosphorylation of STAT5A, even though PKM2 physically binds with STAT5A. In addition, S202D mutant PKM2 retained pyruvate kinase activity that was as high as wild-type PKM2 (Supplementary Figure S5). These results suggest that p-S202-PKM2 may function as a metabolic enzyme to provide ATP or as a cofactor for the transcriptional activation of STAT5A, similar to its function in the HIF-1 system [28]. Thus, the physiological function of nuclear PKM2 under IGF stimulation should be further explored.

In conclusion, we demonstrated that PKM2 expression is crucial for the IGF-1-dependent activation of cancer cell growth, and that AKT directly binds with PKM2 to phosphorylate it at Ser-202. We demonstrated that this phosphorylation is necessary for the nuclear translocation of PKM2 protein and, subsequently, its binding with STAT5 under IGF stimulation, which is critical for IGF-1-stimulated cancer cell growth. Collectively, our data demonstrate a molecular mechanism of IGF-1 signaling in the context of altered tumor metabolism and support a crucial function of PKM2 for promoting the growth of cancer cells with aberrantly activated IGF-1/PI3K/AKT signaling.

## MATERIALS AND METHODS

### Cells and reagents

The non-small cell lung carcinoma cell line NCI-H1299 and the human embryonic kidney cell line HEK293T were purchased from American Type Culture Collection (ATCC, Rockville, MD, USA) and cultured with RPMI 1640 or Dulbecco's modified Eagle medium, respectively, supplemented with 10% fetal bovine serum (FBS; Gibco, Grand Island, NY, USA) at 37°C in a humidified incubator with 5% CO_2_. IGF-1 (291-G1; R&D Systems, Minneapolis, MN, USA) was used at a concentration of 200 ng/ml. Lipofectamine and Lipofectamine RNAi MAX reagents (Life Technologies, Carlsbad, CA, USA) were utilized for transfection of DNA vectors or siRNAs, respectively. Anti-PKM2 antibody (4053S) was purchased from Cell Signaling Technology (Beverly, MA, USA); anti-STAT5 (sc-835), anti-CCND1 (sc-20044), anti-STAT5A (sc-1081), and anti-p-STAT5 (sc-11761) antibodies were obtained from Santa Cruz Biotechnology (Santa Cruz, CA, USA); and anti-p-Ser (ab9332), anti-p-Thr antibodies (ab9337) were from Abcam (Cambridge, UK). The MEK inhibitor PD098059 (P215) and the AKT inhibitor (A6730) were purchased from Sigma (St. Louis., MO, USA). Recombinant PKM2 protein was purchased from BioVision (Mountain View, CA, USA).

### Cell proliferation assay

Cells were seeded at 2 × 10^4^ cells per plate on 60-mm dishes, incubated at 37°C for 24 h, and then the media were replaced with fresh media containing 0.5% FBS. After 24 h, the cells were incubated with or without IGF-1 (200 ng/ml) for 3 days. Cell numbers were measured with an automated cell counter (Bio-RAD, TC-10). All experiments were performed in triplicate.

### Subcellular fractionation

Cells were suspended with 500 μl of lysis buffer (20 mM HEPES [pH 7.4], 10 mM KCl, 1.5 mM MgCl_2_, 1 mM EDTA, 1 mM EGTA, 1 mM dithiothreitol, and protease inhibitor cocktail (11873580001); Roche, Mannheim, Germany) containing 250 mM sucrose, and then passed through a 25-G needle 10 times using a 1-ml syringe on ice for 20 min. Nuclei were pelleted at 2,000 × *g* for 5 min. The supernatant was removed into a new tube, centrifuged again at 8,000 × *g* for 5 min, and the supernatant was removed as the cytosolic fraction. The nuclear pellets were lysed by vortexing 3 times for 10 min in the lysis buffer containing 10% glycerol and 0.1% SDS. The nuclear lysates were centrifuged at 8,000 × *g* for 5 min and the supernatants were used as the nuclear fractions.

### Virus preparation and infection

shRNAs against PKM2 (Sigma-Aldrich, USA) or a control shRNA of enhanced green fluorescent protein were co-transfected into HEK293T cells with the *Gag*, *Pol*, and *Env* vectors of lentiviral components. After 24 h, the culture media were collected and filtered with a 0.2-μm pore-size syringe filter (No. 16534K, Goettingen, Germany). H1299 cells were infected with the lentivirus particles and virus-infected cells were selected with 10 mg/ml of puromycin.

### Immuno-purification

H1299 cells were transfected with a Flag-PKM2 vector and, after 24 h, were incubated in a starvation medium containing 0.5% FBS for 24 h. The cells were cultured with or without IGF-1 (200 ng/ml) for 15 min in serum-free media. Cells were lysed with RIPA buffer (20 mM Tris [pH 7.5], 150 mM NaCl, 1 mM EDTA, 5% NP-40, and protease inhibitor cocktail) and centrifuged at 8,000 × *g* for 15 min. The supernatants were subjected to an immunoprecipitation assay using anti-Flag beads. After 12-h incubation at 4°C, the beads were washed 3 times with 1 ml RIPA buffer. Flag-PKM2 was eluted with RIPA buffer containing 0.1 mg/ml Flag peptide.

### *In vitro* kinase assay

A phosphorylation assay was performed in 1X kinase buffer (Cell Signaling Technology, 9802) containing 3.3 μM ATP (Cell Signaling Technology, 9804) with 1 μg of wild-type or S202A mutant GST-PKM2 and 200 ng of active AKT1 (Millipore, 14-276). After incubation for 1 h at 30°C, the reaction was stopped by adding SDS sample buffer and then the samples were subjected to western blot analysis using anti-phospho-serine (Abcam, ab9332) or anti-phospho-threonine (Abcam, ab9337) antibodies. For the autoradiogram analysis, 10 μCi of [γ-32p]ATP (Perkin Elmer, NEG002A250UC) was added to the reaction. The analyzed SDS-PAGE gel was dried and exposed to X-ray film (Kodak) for 3 days.

### LC-MS/MS analysis

Protein bands corresponding to PKM2 were excised from the SDS-PAGE gel and digested in-gel with trypsin for micro-LC-MS/MS analysis. The dried tryptic-digested peptides were resuspended in IMAC binding buffer (40% acetonitrile, 0.1% formic acid). PHOS-Select iron affinity gel was incubated with the digested peptides for 1 h at room temperature. After the beads were washed with 1 ml of the IMAC binding buffer, the bound phosphopeptides were eluted using 200 μl of IMAC elution buffer (200 mM NH_4_H_2_PO_4_). The resulting phosphopeptide samples were analyzed by micro LC-MS/MS on an Agilent HP 1100 quaternary nano-LC system coupled online to an LTQ linear ion trap mass spectrometer (Thermo Finnigan). Buffer A (5% acetonitrile and 0.1% formic acid) and buffer B (80% acetonitrile and 0.1% formic acid) were used to establish a 120-min gradient. The gradient profile started with 100% buffer A, followed by a 100-min gradient from 0% to 50% of buffer B, a 10-min gradient from 50% to 100% of buffer B, and a 10-min gradient of 100% of buffer B. Data-dependent scans consisting of one full MS scan (400–1,400 m/z) and 10 data-dependent MS/MS scans were used to generate MS/MS spectra of the eluted peptides. MS/MS spectra were searched against an in-house database containing various PKM2 homologue sequences using SEQUEST (http://fields.scripps.edu/sequest). The variable modifications applied to the search were methionine oxidation and the phosphorylation of serine, threonine, and tyrosine, and the carboxyamidomethylation of cysteine was set as a fixed modification. DTAselect was used to filter the search results with the following criteria: Xcorr > 1.5 for charge state 1+, Xcorr > 2.0 for charge state 2+, Xcorr > 3.0 for charge state 3+, and fully tryptic digest end requirement. Assignments of the phosphorylation site were finally confirmed by manual validations on the filtered MS/MS spectra.

### STAT5 reporter assay

Firefly and *Renilla* luciferase activities were performed according to the manufacturer's manual (Promega, Madison, WI, USA). Cells (1 × 10^4^ per well on 12-well plate) were transfected with pGL4.52 (luc2p/STAT5 RE/Hygro) vector (125 ng) and *Renilla* vector (25 ng, internal control), and pcDNA 3.1 (125 ng) or pcDNA-MYC-PKM2 (125 ng) vectors and incubated for 24 h with complete medium. After 24 h, the medium was replaced with fresh medium containing 0.5% serum, and then the cells were treated with IGF-1 (200 ng/ml) for 24 h. The cells were harvested using Promega lysis buffer. Luciferase activities were measured using the substrates provided in the reporter assay system (Promega). The luciferase activity was normalized to *Renilla* luciferase activity.

## SUPPLEMENTARY EXPERIMENTAL PROCEDURE


