# Supplementary material for: AKT-induced PKM2 phosphorylation signals for IGF-1-stimulated cancer cell growth
Source: Oncotarget. 2016 Jun 20;7(30):48155–67. doi: 10.18632/oncotarget.10179 (PMC5217008; doi:10.18632/oncotarget.10179)
Supplement: Supplementary file 1 [file oncotarget-07-48155-s001.pdf]

AKT-induced PKM2 phosphorylation signals for IGF-1-stimulated cancer cell growth

SUPPLEMENTARY EXPERIMENTAL PROCEDURE

In vitro pyruvate kinase activity

Enzymatic activity assay for PKM2 was carried out using the PKM2 Pyruvate Kinase Assay Kit, in accordance with the manufacturer's instructions (BioVision, Milpitas, CA). Briefly, assays were conducted in the presence of recombinant GST-PKM2 protein (100 ng). To determine the enzyme kinetics, the fluorescence of each sample was measured at excitation and emission wavelengths of 530 nm and 590 nm, respectively, at room temperature at every 10-minute for 1 h.

SUPPLEMENTARY FIGURES

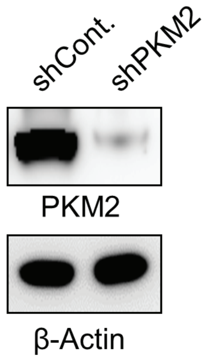

Supplementary Figure S1: Expression level of PKM2 in the stably PKM2-depleted H1299 lung cancer cells. The cells were transduced with shControl or shPKM2 vector using the pLKO lentiviral system. Cell lysates were applied to western blot analysis.

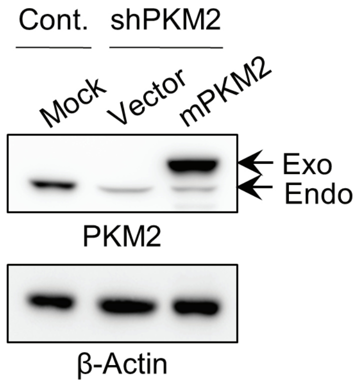

Supplementary Figure S2: Expression level of mouse PKM2 (mPKM2) in the stably PKM2-depleted H1299 lung cancer cells. The PKM2 knockdown cells were transduced with mPKM2-expressing or mock vector. Cell lysates were applied to western blot analysis.

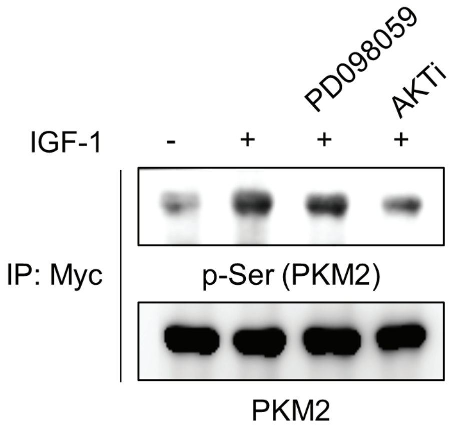

Supplementary Figure S3: Determination of PKM2 phosphorylation on serine residues under IGF-1 stimulation. H1299 cells were transfected with Myc-PKM2 vector and, after 24 h, starved under media containing 0.5% FBS for a further 24 h. Cells were pretreated with PD098059 (10 µM), AKTi (10 µM), or dimethyl sulfoxide for 30min and then treated with or without IGF-1 (200 ng/ml) for 30 min. Cells were harvested and subjected to an immunoprecipitation assay using anti-Myc antibody for Myc-tagged PKM2.

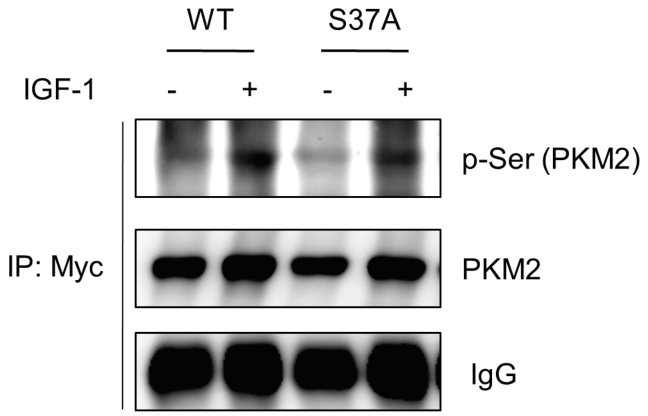

Supplementary Figure S4: Analysis of the phosphorylation of the PKM2(S37A) mutant at serine residue(s) under IGF-1 stimulation. H1299 cells were transfected with the MYC-tagged wild-type (WT) PKM2 or S37A mutant PKM2 vector. After treatment with or without IGF-1 (200 ng/ml) for 30 min, cell lysates were prepared and subjected to an immunoprecipitation assay using anti-MYC antibody. The level of phospho-Ser PKM2 was determined using anti-p-Ser antibody.

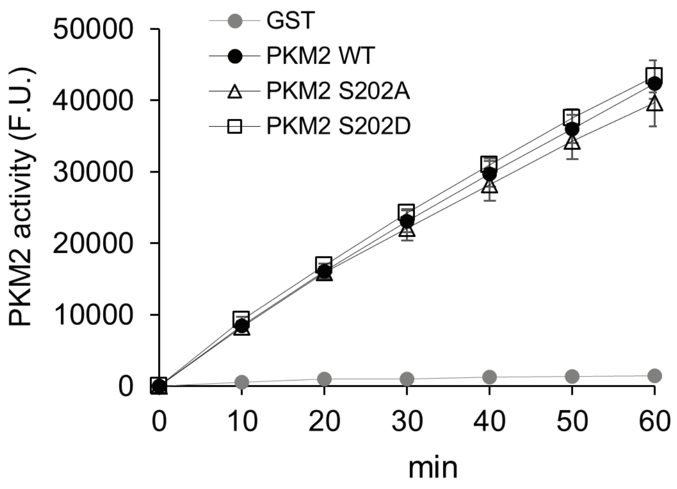

Supplementary Figure S5: Determination of the pyruvate kinase activity of PKM2 wild-type or mutants. ATP assay was carried out using the Pyruvate Kinase (ATP Colorimetric/Fluorometric) Assay Kit in the presence (100 ng) of each recombinant GST-fused PKM2 protein. The fluorescence of each sample was measured at excitation and emission wavelengths of 530 nm and 590 nm, respectively. All data shows the mean ±s.d. of three individual experiments.

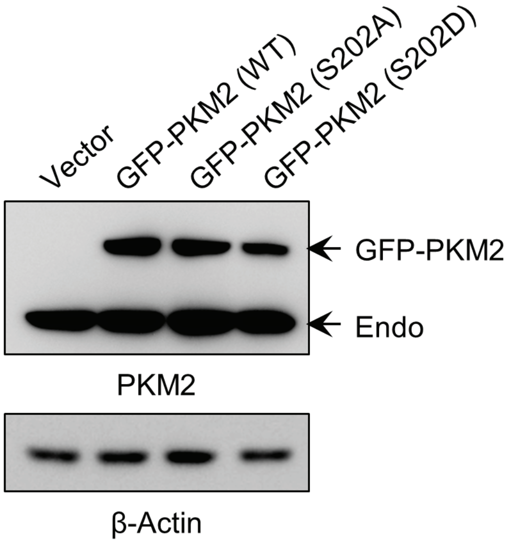

Supplementary Figure S6: Expression of GFP-PKM2 proteins in the H1299 lung cancer cells. The cells were transduced with GFP-fused PKM2 wild-type or S202 mutant vectors.

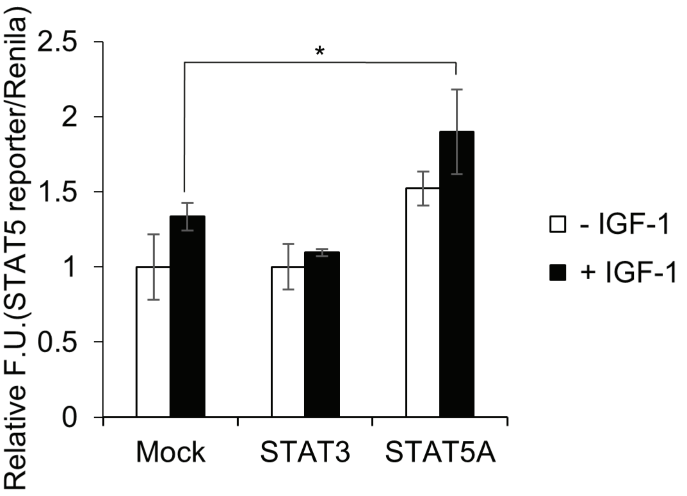

Supplementary Figure S7: Determination of the STAT5 specificity of the STAT5 reporter system. Cells were transfected with the pcDNA, pcDNA-STAT3, or pcDNA-STAT5A vector together with the STAT5 reporter system vectors. The results were obtained from two independent experiments performed in triplicate.

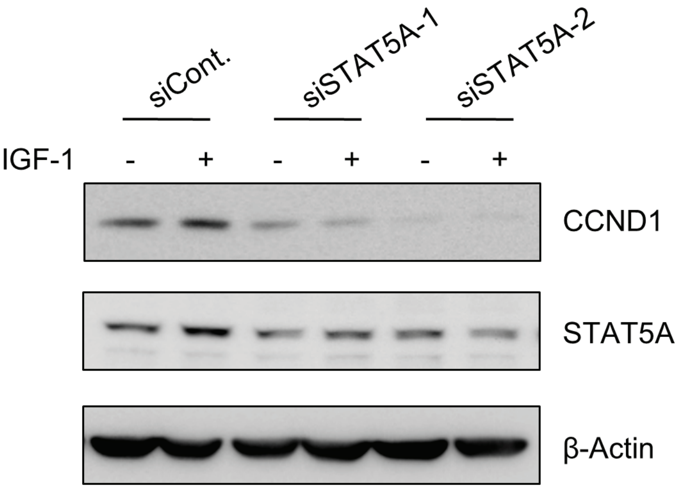

Supplementary Figure S8: Expression of CCND1 in H1299 cells. H1299 cells were transfected with two siSTAT5A sequences individually. After 24 h, the cells were starved with media containing 0.5% FBS for 24h, and then treated IGF-1 for 24 h. Cells were harvested and applied to western blot analysis, and the results were visualized with CCND1 or STAT5A antibody.

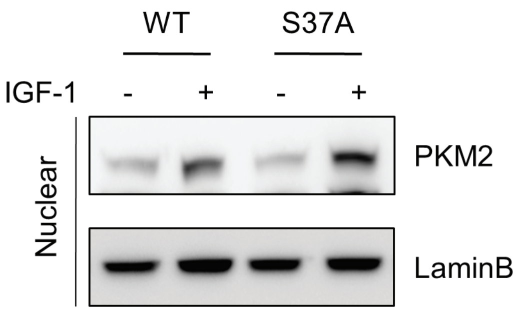

Supplementary Figure S9: Analysis of the nuclear localization of the PKM2(S37A) mutant at serine residue(s) under IGF-1 stimulation. H1299 cells were transfected with the MYC-tagged wild-type (WT) PKM2 or S37A mutant PKM2 vector. After treatment with or without IGF-1 (200 ng/ml) for 12 h, the nuclear fraction was extracted and subjected to western blot analysis with anti-PKM2 antibody.
